# Defining clinical pharmacy and support activities indicators for hospital practice using a combined nominal and focus group technique

**DOI:** 10.1007/s11096-021-01298-z

**Published:** 2021-06-24

**Authors:** Hugo Lopes, Andrea Rodrigues Lopes, Helena Farinha, Ana Paula Martins

**Affiliations:** 1 IQVIA, Lisbon, Portugal; 2 NOVA National School of Public Health, Public Health Research Centre, Universidade NOVA de Lisboa, Lisbon, Portugal; 3 Comprehensive Health Research Center (CHRC), Universidade NOVA de Lisboa, Lisbon, Portugal; 4 Pharmaceutical Society Board Member, Lisbon, Portugal; 5 Pharmacy Department, Hospital Egas Moniz Coordinator, Centro Hospitalar de Lisboa Ocidental, Lisbon, Portugal; 6 Faculty of Pharmacy, University of Lisbon, Lisbon, Portugal

**Keywords:** Hospital pharmacy, Key performance indicators, Portugal

## Abstract

*Background *Although clinical pharmacy is a crucial part of hospital pharmacist’s day-to-day activity, its performance is not usually subject to a holistic assessment. *Objective* To define a set of relevant and measurable clinical pharmacy and support activities key performance indicators (cpKPI and saKPI, respectively). *Setting* Portuguese Hospital Pharmacies. *Method* After a comprehensive literature review focusing on the metrics already in use in other countries, several meetings with directors of hospital pharmacies were conducted to obtain their perspectives on hospital pharmacy practices and existing metrics. Finally, five rounds with a panel of 8 experts were performed to define the final set of KPIs, where experts were asked to score each indicator’ relevance and measurability, and encouraged to suggest new metrics. *Main outcome measure* The first Portuguese list of KPIs to assess pharmacists’ clinical and support activities performance and quality in hospital pharmacies. *Results* A total of 136 KPIs were assessed during this study, of which 57 were included in the original list and 79 were later added by the expert panel. By the end of the study, a total of 85 indicators were included in the final list, of which 40 are considered to be saKPI, 39 cpKPI and 6 neither. *Conclusion* A set of measurable KPIs was established to allow for benchmarking within and between Portuguese hospital Pharmacies and to elevate professional accountability and transparency. Future perspectives include the use of both cpKPIs and saKPIs on a national scale to identify the most efficient performances and areas of possible improvement.

## Impacts on practice

- This work intends to promote the discussion around performance indicators and to raise awareness and know-how on the current and future role of the Hospital Pharmacies, by determining a framework to develop it assessment on a recurrent basis.
- For the first time in Portugal, a set of relevant and measurable indicators are defined in order to assess hospitals’ pharmacies performance, using a combined nominal group/focus group technique.
- The definition of these performance indicators is considered to be a landmark in hospital pharmacy in Portugal and it is the first step to the first national study to assess all hospital pharmacies of the NHS.
- Since an international benchmarking system is not established for hospital pharmacies, it is essential to cultivate an internal culture of activity monitoring and internal benchmarking between similar institutions, using a set of KPI defined by hospital pharmacists, based on the evidence available.

## Introduction

Hospital pharmacists strive to continuously maintain and improve medication management and patient pharmaceutical care to the highest possible standards. Their roles include participating in medication management, which encompasses the entire way in which medicines are selected, procured, delivered, prescribed, administered and monitored [1, 2]. These activities are performed whilst ensuring the 7 “rights” are respected: right patient, right dose, right route, right time, and the right drug with the right information and documentation.

Clinical pharmacy is defined as “a health science discipline where pharmacists provide patient care that optimizes medication therapy and promotes health, wellness, and disease prevention” [3–5]. Therefore, clinical pharmacy is deemed an integral component of this process, being responsible for ensuring that patients receive the right medicine at the right time by an efficient and economic system [6].

Although traditionally pharmacists were mostly concerned with procuring, dispensing, manufacturing and supplying drugs [2], clinical pharmacy has become so relevant that pharmacists spent an average of 47% of their time on clinical activities, 37% on distribution and 16% on management activities, as shown by an Australian study [7].

In fact, in Portugal, as in other countries, the hospital pharmacy concept lies in the existence of two major areas: support sector and clinical activities [8]. The first integrates management and organization, acquisition and stock management, storage and conservation, repackaging, production/compounding, and distribution. The clinical area involves all the activities related to clinical pharmacy/pharmaceutical care (e.g., therapeutic review, medication reconciliation, pharmaceutical consulting, clinical pharmacokinetics, counselling or pharmacovigilance).

In Portugal, the pharmaceutical profession emerged in the thirteenth century [9]**.** Out of 15,000 practicing pharmacists, 9% are in hospital pharmacies [8]. Given the evolution of healthcare and patient’s needs and demands, the 2008 Hospital Medicine Program allowed hospital pharmacists to participate and develop quality improvement initiatives, promoting a patient safety culture [8, 10–12]

Evidence suggests that when clinical pharmacists integrate the multidisciplinary team, their interventions can help reduce the likelihood of mortality, length of stay, adverse-drug-event prevalence and improve patients’ quality of life [6, 16], by ensuring medication reconciliations/reviews [5, 13–15]. Therefore, a way to assess both quality and impact of the services provided to patients is by quantifying and monitoring clinical activities through audits, service reviews, incident reports and surveys to patients, and by ensuring that complaint management and control procedures are in place [5, 13, 17] .

A known strategy to track and continuously assess performance is through the use of clinical pharmacy Key Performance Indicators (cpKPIs) [6]. According to several studies, cpKPIs could be used to evaluate the quality of care [17–19], to help define a patients’ healthcare expectations regarding a clinical pharmacist, to allow benchmarking within and between organizations, to elevate professional accountability and transparency [5], and to allow the tracking of the organization’s progress towards achieving predefined goals and standards of care [4, 5]. They also play an important part in rewarding good performance, in improving resource allocation and efficiency, and in identifying and reducing clinical errors, whilst maximizing healthcare outcomes and balancing patient’s wants and needs [17–20]. Given the wide range of services provided, assessing pharmacists’ productivity and quality of care is somewhat difficult [21, 22]. Thus, it is also relevant to establish KPIs addressed to support activities (saKPI).

Despite the evidence supporting the importance of defining KPIs to quantify pharmacists contribution to patient care [5, 19, 20, 23–26], three main barriers were identified by several authors regarding their implementation: (i) resistance to change related to documenting clinical activities due to increased workload, practice environment constraints and competing priorities; (ii) disbelief of KPIs’ real benefits, value and existing support from other pharmacists and hospital administrations; and, (iii) uncertainty of how to address quality versus quantity or the influence KPIs may have in the future of pharmacy practice [6, 27–29].

Nevertheless, several countries have already started developing their own standard KPIs, such as Australia [13, 26], 30–33], Belgium [19, 25], Brazil [20, 34, 35], Canada [4, 5, 36, 37], Finland [38], Spain [39, 40], UK [17], USA [41–44], New Zealand [22, 45], and the Netherlands [46]. However, there is no current international consensus on KPIs [2, 4–6, 13, 21, 29].

### Aim of the study

In Portugal, most hospital pharmacies only collect some internal data for certification/accreditation purposes or *ad-hoc* situations. Currently, there is no national standard system for activity monitoring, nor any nationwide framework that enables comparisons/benchmarks amongst pharmacies’ performances regarding their clinical or support activities. Thus, the main goal of this study is to define, for the first time in Portugal, a national set of relevant and measurable cpKPIs/saKPIs to assess the National Health System Hospital Pharmacies’ performance and quality.

### Ethics approval

As a service development and evaluation study, it was exempt from formal ethics approval. All study participants were given full information and provided signed, informed consent.

## Method

### Study design

**Fig. 1:**
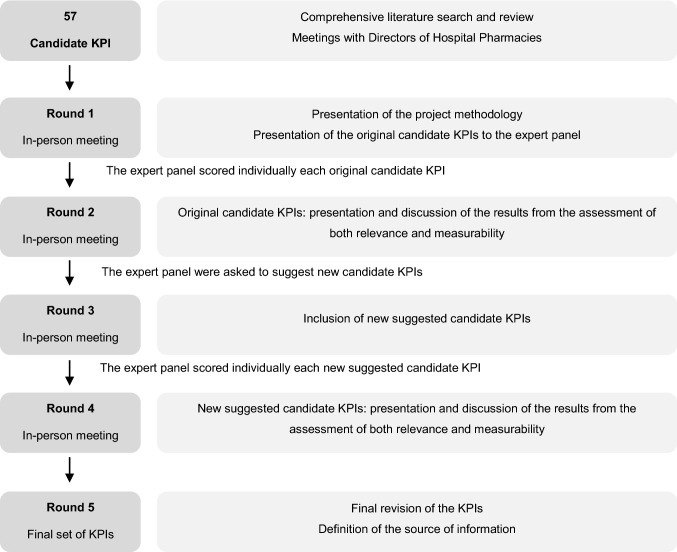
Study methodology

Consensus cpKPI/saKPI were determined using a combined nominal group/focus group technique, which combines the prioritisation process of a standard nominal group technique with the in-depth discussion of a focus group [47]. The expert panel was encouraged to assess both the relevance and measurability of the original candidate KPI, and to suggest new candidate indicators, for five rounds. After each round, an in-person panel meeting was held, promoting in-group discussions about the candidate KPIs and to clarify questions regarding the definition of any new proposed KPI (Fig. 1).

### Defining the KPIs

Two stages were developed to define the first list of cpKPI/saKPI: (1) Literature review and exploratory meetings; (2) Expert panel rounds.

1. Literature review and exploratory meetings

Two investigators (HL and ARL) conducted a comprehensive literature review focusing on KPIs used in other countries. To account for existing national practices, meetings were held with several renown hospital pharmacists, who shared their perspectives on currently implemented practices and metrics. After this process, a list of 57 candidate KPIs was defined.

The annual European Association of Hospital Pharmacists (EAHP) survey is annually deployed to measure the progress, key barriers and drivers of the implementation of their six Statements: (i) Introductory Statements and Governance; (ii) Selection, Procurement and Distribution; (iii) Production and Compounding; (iv) Clinical pharmacy Services; (v) Patient Safety and Quality Assurance; and, (vi) Education and Research. Since several Portuguese hospital pharmacies already participate in the survey [1, 48], each EAHP Statement was divided into several Assessment Areas and specific candidate KPIs were defined to assess each Area.

(2) Expert panel rounds

To reach the final list of KPIs, five rounds with an expert panel were performed from January of 2019 to March of 2020.

#### Round 1

The first round presented to the expert panel the key project moments, the main goals and methodology, all of which previously defined with the Portuguese Pharmaceutical Society, and the 57 candidate KPIs, categorised by EAHP Statement.

After this round, the expert panel had four weeks to rate each indicator in two different dimensions: Relevance and Measurability. The former was defined as the ability to reflect the hospital pharmacy performance or the clinical pharmacist’s direct impact on patient care, while the latter was defined as the ability to easily collect data to calculate the KPI within the hospital.

Each panel member used a five-point Likert scale to assess both dimensions: 1 = Totally Irrelevant/Totally Impossible; 2 = Not Relevant/Impossible; 3 = Neutral/Neutral; 4 = Relevant/Easy; 5 = Very Relevant/Very Easy.

At the beginning of the study, three criteria were defined by the expert panel to determine which KPIs would be included in the final list: (i) if the average *relevance* score for each indicator was low (rating equal or lower than 3 points), the indicator would be excluded, regardless the *measurability* score; (ii) if the average *relevance* score was high (rating higher than 3 points) and the *measurability* low, the indicator would be excluded; and, (iii) if the average *relevance* and *measurability* scores were high, the indicator would be included in the final list.

#### Round 2

After collecting all panellist scores and calculating the average score for each indicator, a second in-person meeting was held to present and discuss the results regarding relevance and measurability of the original candidate KPIs. In each in-person meeting, consensus concerning indicators with scores close to cut-off points were obtained by majority.

#### Round 3

The expert panel was given four weeks to suggest new candidate KPIs per EAHP Statement. Following this time, a third in-person panellist meeting was held for discussion and clarification regarding the proposed KPIs and their definitions.

#### Round 4

The expert panel was then asked to rate the new set of suggested KPIs according to their relevance and measurability, using the five-point Likert Scale. The fourth in-person round took place after having all the scores calculated for each indicator.

#### Round 5: Final set of KPIs

Finally, after assessing both original and suggested candidate KPIs, a last in-person meeting was held to present the final list and to define the sources of information available within the hospitals to measure each KPI.

### KPI Definition

Concerning the definition of the candidate KPIs, the authors agreed that all should: (i) reflect the current hospital pharmacists activities, (ii) be evidence-based, (iii) be aligned with clinical pharmacists’ goals, objectives and practices, (iv) be feasible to measure, (v) be relevant to clinical outcomes, and (vi) be used across all types of Hospital Pharmacies (e.g., rural, urban, teaching or non-teaching hospitals). A glossary indicating each rational, measurement unit, target-population and data-source was then prepared for each suggested candidate KPI.

#### The expert panel

A panel of eight experts was specifically selected for this project by the board members of the Portuguese Pharmaceutical Society, considering their professional curricula, expertise, and contributions for the development of clinical pharmacy in Portugal. These experts are renowned hospital pharmacists having also professional responsibilities since they are Pharmaceutical Society representatives and members of pharmaceutical associations in Portugal.

As for their main characteristics, the average age was 48.7 years old, mostly females, with around 25 years of experience as a pharmacist and around 10 years of experience as hospital pharmacy director (Table 1).

**Table 1:** Expert panel characteristics

Characteristics		
Number of experts	N	8
Age	Mean (SD)	48.7 (5.4)
Gender female	N (%)	6 (75%)
Years of experience as pharmacist	Mean (SD)	24.5 (5.7)
Level of education	Post-graduation degree: N (%)	4 (50%)
	Master’s degree: N (%)	3 (38%)
Specialist title (yes)	N (%)	8 (100%)
Years as hospital pharmacy director	Mean (SD)	10.2 (5.3)
Member of hospital pharmacy commission (yes)	N (%)	6 (75%)
Years as member of hospital pharmacy commission	Mean (SD)	12.3 (6.0)

The definition of a panel consisting exclusively of pharmacists aims to ensure that the defined cpKPIs/saKPIs are unanimously agreed upon, and that they effectively measure their performance. In an analogy of the Gettysburg Address speech by former U.S. President Abraham Lincoln, this expert panel was created to define the cpKPI/saKPI list for Hospital Pharmacy “of the pharmacists, by the pharmacists, for the pharmacists”.

## Results

### Round 1 and 2: assessing the original list of the candidate KPIs

Following an extensive literature review, 57 candidate KPIs were included in the original list, categorized into six EAHP Statements (Table 2), where 22 were considered as saKPI, 33 cpKPI and 2 neither.

**Table 2:** Assessing the original list of the candidate KPI (Round 1 and 2)

EAHP Standards	Assessment area	Key performance indicators	Type of KPI	Relevance (average)	Measurability (average)	Final list
I. Statement of Introductory Principles and Management	Human resources	Number of Full Time Equivalent (FTE) Professionals, adjusted by number of beds	saKPI	4.7	4.7	Y
	Human resources	Burden of absenteeism hours by pharmacist FTE	saKPI	4.3	4.5	Y
	Technology/Software	Existence of an electronic prescription system integrated with the pharmacy (Identify in which production lines)	cpKPI	4.3	4.8	Y
	Technology/Software	Existence of a double-check medication repackaging system	saKPI	4.3	4.3	Y
	Technology/Software	Existence of a double-check system in the production / compounding of sterile products	saKPI	4.5	4.7	Y
	Technology/Software	Existence of a double-check system in the production / compounding of non-sterile products	saKPI	4.5	4.7	Y
	Certifications/Accreditations	Existence of a quality management system	saKPI	3.4	2.9	N
	Technology/Software	Pharmacists routinely used a mobile device while providing patient care	cpKPI	2.3	2.0	N
	Technology/Software	Pharmacy track and monitor trends in financial metrics	saKPI	2.4	3.1	N
II. Selection, procurement and distribution	Inventory and logistics management	Drugs stock turnover rate (in days)	saKPI	4.2	4.0	Y
	Drug distribution	Existence of an automated inpatient medication preparation system (which one?)	saKPI	4.5	4.5	Y
	Drug distribution	Existence of an automated outpatient medication distribution system (which one?)	saKPI	4.5	4.5	Y
	Drug distribution	Number of drugs dispensed to outpatients	cpKPI	4.3	4.2	Y
	Drug distribution	Existence of an automated inpatient distribution system (which one?)	saKPI	4.5	4.5	Y
	Drug distribution	Existence of an automated outpatient dispensing system (which one?)	saKPI	4.5	4.5	Y
	Drug distribution	Percentage of hospital beds in Unit Dose	saKPI	4.7	4.5	Y
	Inventory and logistics management	Drugs obsolescence rate (lost due to expiry date)	saKPI	4.3	2.5	N
	Drug distribution	Total inpatient doses dispensed per number of inpatient discharges	saKPI	3.5	2.8	N
	Drug distribution	Total inpatient doses returned	saKPI	4.3	2.8	N
III. Production and preparation	Drug preparations	Ability to prepare internally sterile and injectable preparation blends	saKPI	4.8	2.6	N
	Drug preparations	Number of biological controls performed	saKPI	4.8	2.0	N
	Drug preparations	Number of sterile and injectable preparation blends performed	saKPI	4.8	2.0	N
IV. Clinical Pharmacy services	Prescription review and reconciliation	Number of inpatient prescriptions validations (medication review), adjusted by pharmacist FTE	cpKPI	5.0	4.3	Y
	Prescription review and reconciliation	Number of outpatient prescription validations (medication review), adjusted by pharmacist FTE	cpKPI	4.5	4.5	Y
	Prescription review and reconciliation	Number of pharmacist interventions in patient therapy, adjusted by pharmacist FTE	cpKPI	4.5	4.5	Y
	Prescription review and reconciliation	Number of blood products dispensed, per 1000 patients discharged	saKPI	4.4	4.3	Y
	Prescription review and reconciliation	Number of narcotic and psychotropic requests analysed, per 1000 patients discharged	cpKPI	4.3	4.2	Y
	Rounds	Number of pharmacists rounds	cpKPI	4.7	2.1	N
	Prescription review and reconciliation	Average of admitted days that patients receive medication review by a pharmacist	cpKPI	4.0	1.5	N
	Prescription review and reconciliation	Proportion of patients for whom pharmacists participate in interprofessional patient care rounds to improve medication management	cpKPI	4.5	1.5	N
	Prescription review and reconciliation	Number of medication reconciliations up to 72 h after admission	cpKPI	4.5	1.5	N
	Prescription review and reconciliation	Number of medication reconciliations at discharge	cpKPI	4.5	1.5	N
	Outpatient activity	Number of outpatient’s pharmaceutical consultations/appointments	cpKPI	4.5	2.1	N
	Information sharing	Number of patients with written information regarding prescribed medications at discharge	cpKPI	4.5	1.2	N
	Information sharing	Number of outpatients with written information regarding prescribed medications	cpKPI	4.5	1.2	N
	Information sharing	The percentage of patients satisfied with the information they received about their medications while in hospital	cpKPI	4.0	1.3	N
V. Patient safety and quality assurance	The seven rights (patient, medication, dose, route, time, information and documentation)	Existence of inpatient pharmacokinetic monitoring protocols (yes / no)	cpKPI	4.8	4.7	Y
	The seven rights (patient, medication, dose, route, time, information and documentation)	Existence of outpatient pharmacokinetic monitoring protocols (yes / no)	cpKPI	4.8	4.7	Y
	Strategies to identify and reduce errors	Rate of nonconformities in total number of internal audits	cpKPI	4.5	4.0	Y
	Strategies to identify and reduce errors	Rate of nonconformities in total number of external audits	cpKPI	4.5	4.0	Y
	Monitoring and reporting of adverse events	Rate of patients with medication errors (reported events), per 1000 patients discharged	cpKPI	5.0	4.5	Y
	High-risk drug management	Existence of an active pharmacovigilance system (yes / no)	cpKPI	5.0	4.3	Y
	High-risk drug management	Number of active pharmacovigilance follow-ups performed	cpKPI	4.6	4.3	Y
	The seven rights (patient, medication, dose, route, time, information and documentation)	Proportion of patients at high risk of venous thromboembolism that receive appropriate prophylaxis	cpKPI	4.5	1.7	N
	The seven rights (patient, medication, dose, route, time, information and documentation)	Percentage of patients interviewed by a pharmacist by the end of the following working day after admission	cpKPI	4.0	2.1	N
	Monitoring and reporting of adverse events	Number of adverse events reported by staff	cpKPI	4.5	2.1	N
	Monitoring and reporting of adverse events	Number of adverse events reported by patients	cpKPI	4.0	1.5	N
	Monitoring and reporting of adverse events	Number of medication errors reported by staff	cpKPI	4.5	3.0	N
	Monitoring and reporting of adverse events	Number of medication errors reported by patients	cpKPI	4.0	1.5	N
	Monitoring and reporting of adverse events	Number of pharmacy attributable events (storage, ordering, administration, preparation/dispense, monitoring)	saKPI	4.0	1.5	N
VI. Education and research	Research and publications	Number of national peer-reviewed publication, adjusted by pharmacist FTE	n.a	4.5	4.0	Y
	Research and publications	Number of international peer-reviewed publication, adjusted by pharmacist FTE	n.a	4.7	4.0	Y
	Clinical trials participation	Number of clinical trials involving hospital pharmacists	cpKPI	5.0	4.5	Y
	Clinical trials participation	Number of clinical trials involving hospital pharmacists, adjusted by pharmacist FTE	cpKPI	5.0	4.5	Y
	Clinical trials participation	Existence of a standardized process for implementation and follow-up of clinical trials	saKPI	5.0	4.5	Y
	Clinical trials participation	Number of patients in clinical trials in which the pharmacist is involved, adjusted by pharmacist FTE	cpKPI	4.8	4.3	Y
	Clinical trials participation	Experimental medication dispensing error rate	cpKPI	5.0	4.0	Y

*EAHP* European association of hospital pharmacists, *saKPI* support activity key performance indicator, *cpKPI* clinical pharmacy key performance indicator, *FTE* full time equivalent, *Y* yes, *N* no, *n.a.* not applicable, Relevance and Measurability scores ranged from 1–5 points

After assessing their *relevance*, only two were considered as “not relevant” (rating lower than 3 points) by the expert panel and therefore were excluded.

Regarding *measurability*, although 21 KPIs were considered *relevant* (rating equal or higher than 4 points), data collection capability was low. For example, the ‘*Number of pharmacists rounds*’ or the ‘*Number of adverse events reported by patients*’ were considered as some of the most relevant KPIs, however, the ability to measure them ranged from 1.5 to 2.1 points. Similarly, the three KPIs rated as ‘Totally Impossible’ to measure (rating equal or lower than 1.3 points) were also considered to be highly relevant (rating equal or higher than 4 points).

### Rounds 3 and 4: expert panel suggested candidate KPIs

The following rounds sought not only to include the new suggested candidate KPIs (round 3), but also to assess their relevance and measurability (round 4) (Table 3).

**Table 3:** Suggested candidates KPI by the expert panel (Round 3 and 4)

EAHP Standards	Assessment area	Key performance indicators	Type of KPI	Relevance (average)	Measurability (average)	Final list
I. Statement of introductory principles and management	Certifications/Accreditations	Pharmacy Certification (which one? number of cycles)	saKPI	5.0	5.0	Y
	Certifications/Accreditations	Pharmacy Accreditation (which one? number of cycles)	saKPI	5.0	5.0	Y
	Human resources	Ratio between pharmacists and technicians FTEs	saKPI	4.3	5.0	Y
	Human resources	Number of postgraduate pharmacists	saKPI	4.2	4.5	Y
	Human resources	Number of pharmacists with a master's or a PhD degree	saKPI	4.2	4.5	Y
	Human resources	Ratio between Specialists Pharmacists and total of Pharmacists	saKPI	4.4	4.5	Y
	Pharmacy committee	Existence of equal representation in the Therapeutic Pharmacy Committee (Identify TPC composition)	cpKPI	5.0	5.0	Y
	Pharmacy committee	Number of drugs introduced in the Health Technology Assessment Information System (SiATS), adjusted by pharmacist FTE	cpKPI	4.3	4.3	Y
	Human resources	Number of professionals with performance evaluation in the year	saKPI	3.5	2.3	N
	Pharmacy committee	Number of monthly meetings of the Therapeutic Pharmacy Committee	cpKPI	3.9	2.2	N
	Pharmacy committee	Rate of medication prescriptions requiring justification and opinion deliberation of the Therapeutic Pharmacy Committee	cpKPI	4.5	2.7	N
	Pharmacy committee	Rate of new medicines and another products introduction	cpKPI	4.3	2.7	N
II. Selection, procurement and distribution	Medication form	Ratio between biosimilar and biological medications	saKPI	4.3	4.0	Y
	Drug distribution	Percentage of hospital beds in Pyxis	saKPI	4.7	4.5	Y
	Drug distribution	Number of Special Use Authorizations, adjusted by pharmacist FTE	cpKPI	4.8	4.0	Y
	Medication form	Existence of a medication form (yes / no)	saKPI	2.5	4.7	N
	Medication form	Number of orders placed outside the medication form	saKPI	3.0	4.0	N
	Inventory and logistics management	Percentage of drugs with an expiration date < 1 month	saKPI	3.8	2.5	N
	Inventory and logistics management	Average time to stock-out of essential medications	saKPI	4.2	2.6	N
	Drug distribution	Number of special use authorization processes initiated	cpKPI	3.8	2.9	N
III. Production and preparation	Operating procedures	Existence of Standard Operating Procedures (SOPs) for every time-specific sterile preparation activities	saKPI	4.8	4.7	Y
	Operating procedures	Existence of Standard Operating Procedures (SOPs) for every time-specific non-sterile preparation activities	saKPI	4.8	4.7	Y
	Facilities and staff	Number of preparations performed in outsourcing, adjusted by pharmacy FTEs	saKPI	4.8	4.5	Y
	Drug preparations	Percentage of contaminations from biological controls	saKPI	4.5	4.2	Y
	Drug preparations	Number of non-sterile preparations, per 1000 patients discharged	saKPI	4.5	4.2	Y
	Drug preparations	Number of sterile preparations, per 1000 patients discharged	saKPI	4.5	4.2	Y
	Facilities and staff	Existence of conditions in hospital for the development of sterile preparations (parenteral nutrition; Chemotherapy or other IV mixtures)	saKPI	2.9	2.9	N
	Drug preparations	Number of contaminated preparations	saKPI	4.2	2.6	N
	Drug preparations	Number of patients discharged with parenteral nutritional preparations and in follow-up	cpKPI	4.1	2.4	N
	Facilities and staff	Existence of conditions in hospital for the development of non-sterile preparations (oral suspensions, syrups, ointments, etc.)	saKPI	2.8	2.8	N
	Drug preparations	Ability to prepare internally requested biological controls	saKPI	4.8	2.7	N
	Drug preparations	Use of operator sleeve control when handling cytotoxic drugs (CTX)	saKPI	2.9	4.0	N
IV. Clinical pharmacy services	Rounds	Percentage of services with pharmacist rounds	cpKPI	4.8	4.0	Y
	Prescription review and reconciliation	Number of inpatients with therapeutic reconciliation, adjusted by pharmacist FTE	cpKPI	4.6	4.0	Y
	Prescription review and reconciliation	Existence of medication reconciliations up to 72 h after admission (yes / no)	cpKPI	4.8	4.0	Y
	Prescription review and reconciliation	Existence of medication reconciliations at discharge (Yes / No)	cpKPI	4.8	4.0	Y
	Prescription review and reconciliation	Number of blood products orders analysed, per 1000 patients discharged	cpKPI	4.5	4.0	Y
	Prescription review and reconciliation	Number of blood products returned per 1000 patients discharged	saKPI	4.4	4.0	Y
	Prescription review and reconciliation	Number of narcotic and psychotropic requests dispensed, per 1000 patients discharged	saKPI	4.4	4	Y
	Outpatient activity	Existence of specific outpatient pharmaceutical consultations (Identify which specialties)	cpKPI	4.8	4	Y
	Outpatient activity	Number of outpatient pharmaceutical consultations, adjusted by pharmacist FTE	cpKPI	4.5	4.2	Y
	Information sharing	Existence of written information regarding prescribed medications at discharge (yes / no)	cpKPI	4.8	4.0	Y
	Information sharing	Existence of written information regarding outpatients prescribed medications (yes / no)	cpKPI	4.8	4.0	Y
	Rounds	Percentage of medical visits accompanied by pharmacists	cpKPI	4.5	1.5	N
	Rounds	Number of patient complaints recorded during visits	cpKPI	4.2	1.3	N
	Prescription review and reconciliation	Number of prescriptions analysed, adjusted by pharmacist FTE	cpKPI	4.6	1.6	N
	Prescription review and reconciliation	Number of validated prescriptions, adjusted by pharmacist FTE	cpKPI	4.5	1.6	N
	Prescription review and reconciliation	Number of suggested changes to prescription, adjusted by pharmacist FTE	cpKPI	4.6	1.6	N
V. Patient safety and quality assurance	The seven rights (patient, medication, dose, route, time, information and documentation)	Number of inpatient pharmacokinetics monitorizations, adjusted by pharmacist FTE	cpKPI	4.4	4.2	Y
	The seven rights (patient, medication, dose, route, time, information and documentation)	Rate of inpatient medicines in clinical pharmacokinetics (which ones)	cpKPI	4.5	4.2	Y
	The seven rights (patient, medication, dose, route, time, information and documentation)	Number of outpatient pharmacokinetics monitorization, adjusted by pharmacist FTE	cpKPI	4.4	4.1	Y
	The seven rights (patient, medication, dose, route, time, information and documentation)	Rate of outpatient medicines in clinical pharmacokinetics (which ones)	cpKPI	4.5	4.2	Y
	The seven rights (patient, medication, dose, route, time, information and documentation)	Rate of Serum therapeutic concentrations levels in total concentration levels	cpKPI	4.8	4.2	Y
	The seven rights (patient, medication, dose, route, time, information and documentation)	Antibiotic use rate (3 classes) in Defined Daily Dose (DDD), per 1000 discharged patients	cpKPI	4.3	4.0	Y
	Strategies to identify and reduce errors	Existence of a process that ensures medication batch traceability in outpatient care (yes / no) (which one)	saKPI	4.8	4.5	Y
	Strategies to identify and reduce errors	Existence of a process that ensures traceability of all medication batches on inpatient care (yes / no) (which one)	saKPI	4.8	4.5	Y
	Strategies to identify and reduce errors	Existence of a process that ensures chemotherapy medication batch traceability (yes / no) (which one)	saKPI	4.8	4.5	Y
	Strategies to identify and reduce errors	Existence of a process that ensures blood product medication batch traceability (yes / No) (which one)	saKPI	4.8	4.5	Y
	Strategies to identify and reduce errors	Number of internal clinical audits	saKPI	4.7	4.0	Y
	Strategies to identify and reduce errors	Number of external clinical audits	saKPI	4.5	4.3	Y
	Monitoring and reporting of adverse events	Number of adverse events reported to National Pharmacovigilance System, per 1000 patients discharged and per number of outpatients followed	cpKPI	5.0	5.0	Y
	High-risk drug management	Number of notifications to the National Pharmacovigilance System that results from active pharmacovigilance, adjusted by pharmacist FTE	cpKPI	5.0	5.0	Y
	High-risk drug management	Existence of a list of high-risk medications (yes / no)	saKPI	4.2	4.3	Y
	High-risk drug management	Percentage of medications stored according to the LASA (Look-Alike, Sound-Alike) nomenclature	saKPI	5.0	4.0	Y
	High-risk drug management	Compliance Index of the last audit according to the LASA (Look-Alike, Sound-Alike) nomenclature	saKPI	4.8	4.0	Y
	High-risk drug management	Existence of active medication-related information (yes / no)	cpKPI	4.7	4.5	Y
	The seven rights (patient, medication, dose, route, time, information and documentation)	Number of prescriptions with incorrect dosage	cpKPI	4.6	2.4	N
	The seven rights (patient, medication, dose, route, time, information and documentation)	Number of patients who did not take medication in the last 24 h	cpKPI	4.7	2.1	N
	Strategies to identify and reduce errors	Number of medication incidents per 1000 days	cpKPI	4.6	2.2	N
	Monitoring and reporting of adverse events	Number of hospitalized patients with complete record of allergic reactions within 24 h after admission	cpKPI	4.0	1.5	N
	Monitoring and reporting of adverse events	Number of patients with morbidity resulting from a preventable adverse effect	cpKPI	4.3	1.5	N
	High-risk drug management	Number of therapeutic reconciliations in polymedicated patients with polymedication, adjusted by pharmacist FTE	cpKPI	4.3	1.8	N
VI. Education and research	Education	Number of undergraduate trainees, adjusted by pharmacy FTE	n.a	4.3	4.0	Y
	Education	Number of undergraduate trainees, adjusted by pharmacist FTE	n.a	4.3	4.0	Y
	Education	Number of postgraduate trainees, adjusted by pharmacy FTE	n.a	4.3	4.0	Y
	Education	Number of postgraduate trainees, adjusted by pharmacist FTE	n.a	4.3	4.0	Y
	Continuing education	Time spending on training, adjusted by pharmacy FTE	saKPI	4.2	4.0	Y
	Continuing education	Time spending on training, adjusted by pharmacist FTE	saKPI	4.2	4.0	Y
	Clinical trials participation	Number of new clinical trials involving hospital pharmacists, adjusted by pharmacist FTE	cpKPI	4.8	4.5	Y

*EAHP* European association of hospital pharmacists, *saKPI* support activity key performance indicator, *cpKPI* clinical pharmacy key performance indicator, *FTE* full time equivalent, *Y* yes, *N* no, *n.a.* not applicable, Relevance and Measurability scores ranged from 1–5 points

After the third round, the expert panel suggested 79 new KPIs: 37 saKPI, 38 cpKPI and 4 neither.

Concerning their *relevance* and *measurability* (round 4), six KPIs were considered as totally relevant (rating 5 points) by all panel members and easily measured (rating equal to or higher than 4 points).

After the fourth round, 26 of the total suggested KPIs were excluded, 5 due to their low relevance and 21 due to their low ability to be measured (rating equal or lower than 3.0 points).

### Round 5: defining the final set of KPI

Finally, a last in-person meeting was held with the expert panel to present the final list of KPIs, and to define the sources of information to calculate each metric (round 5).

The expert panel defined a final list with 85 KPIs to assess all six EAHP Statements: 14 to assess the introductory statements; 10 on selection, procurement and distribution; 6 on production and compounding; 16 on clinical pharmacy services; 25 on patient safety and quality assurance; and 14 on education and research (Table 4). Concerning the type of KPI, 40 are saKPIs, 39 cpKPIs and 6 neither.

**Table 4:** Final key performance indicators list (Round 5)

EAHP Standards	Assessment area	Key performance indicators	Type of KPI	Relevance (average)	Measurability (average)	Numb. KPI
I. Statement of introductory principles and management	Certifications/Accreditations	Pharmacy Certification (which one? number of cycles)	saKPI	5.0	5.0	1
	Certifications/Accreditations	Pharmacy Accreditation (which one? number of cycles)	saKPI	5.0	5.0	2
	Human resources	Number of Full Time Equivalent (FTE) Professionals, adjusted by number of beds	saKPI	4.7	4.7	3
	Human resources	Ratio between pharmacists and technicians FTEs	saKPI	4.3	5.0	4
	Human resources	Burden of absenteeism hours by pharmacist FTE	saKPI	4.3	4.5	5
	Human resources	Number of postgraduate pharmacists	saKPI	4.2	4.5	6
	Human resources	Number of pharmacists with a master's or a PhD degree	saKPI	4.2	4.5	7
	Human resources	Ratio between Specialists Pharmacists and total of Pharmacists	saKPI	4.4	4.5	8
	Pharmacy committee	Existence of equal representation in the Therapeutic Pharmacy Committee (Identify TPC composition)	cpKPI	5.0	5.0	9
	Pharmacy committee	Number of drugs introduced in the Health Technology Assessment Information System (SiATS), adjusted by pharmacist FTE	cpKPI	4.3	4.3	10
	Technology/Software	Existence of an electronic prescription system integrated with the pharmacy (Identify in which production lines)	cpKPI	4.3	4.8	11
	Technology/Software	Existence of a double-check medication repackaging system	saKPI	4.3	4.3	12
	Technology/Software	Existence of a double-check system in the production / compounding of sterile products	saKPI	4.5	4.7	13
	Technology/Software	Existence of a double-check system in the production / compounding of non-sterile products	saKPI	4.5	4.7	14
II. Selection, procurement and distribution	Medication form	Ratio between biosimilar and biological medications	saKPI	4.3	4.0	15
	Inventory and logistics management	Drugs stock turnover rate (in days)	saKPI	4.2	4.0	16
	Drug distribution	Existence of an automated inpatient medication preparation system (which one?)	saKPI	4.5	4.5	17
	Drug distribution	Existence of an automated outpatient medication distribution system (which one?)	saKPI	4.5	4.5	18
	Drug distribution	Number of drugs dispensed to outpatients	cpKPI	4.3	4.2	19
	Drug distribution	Existence of an automated inpatient distribution system (which one?)	saKPI	4.5	4.5	20
	Drug distribution	Existence of an automated outpatient dispensing system (which one?)	saKPI	4.5	4.5	21
	Drug distribution	Percentage of hospital beds in Unit Dose	saKPI	4.7	4.5	22
	Drug distribution	Percentage of hospital beds in Pyxis	saKPI	4.7	4.5	23
	Drug distribution	Number of Special Use Authorizations, adjusted by pharmacist FTE	cpKPI	4.8	4.0	24
III. Production and preparation	Operating procedures	Existence of Standard Operating Procedures (SOPs) for every time-specific sterile preparation activities	saKPI	4.8	4.7	25
	Operating procedures	Existence of Standard Operating Procedures (SOPs) for every time-specific non-sterile preparation activities	saKPI	4.8	4.7	26
	Facilities and staff	Number of preparations performed in outsourcing, adjusted by pharmacy FTEs	saKPI	4.8	4.5	27
	Drug preparations	Percentage of contaminations from biological controls	saKPI	4.5	4.2	28
	Drug preparations	Number of non-sterile preparations, per 1000 patients discharged	saKPI	4.5	4.2	29
	Drug preparations	Number of sterile preparations, per 1000 patients discharged	saKPI	4.5	4.2	30
IV. Clinical pharmacy services	Rounds	Percentage of services with pharmacist rounds	cpKPI	4.8	4.0	31
	Prescription review and reconciliation	Number of inpatients with therapeutic reconciliation, adjusted by pharmacist FTE	cpKPI	4.6	4.0	32
	Prescription review and reconciliation	Number of inpatient prescriptions validations (medication review), adjusted by pharmacist FTE	cpKPI	5.0	4.3	33
	Prescription review and reconciliation	Existence of medication reconciliations up to 72 h after admission (yes / no)	cpKPI	4.8	4.0	34
	Prescription review and reconciliation	Existence of medication reconciliations at discharge (Yes / No)	cpKPI	4.8	4.0	35
	Prescription review and reconciliation	Number of outpatient prescription validations (medication review), adjusted by pharmacist FTE	cpKPI	4.5	4.5	36
	Prescription review and reconciliation	Number of pharmacist interventions in patient therapy, adjusted by pharmacist FTE	cpKPI	4.5	4.5	37
	Prescription review and reconciliation	Number of blood products orders analysed, per 1000 patients discharged	cpKPI	4.5	4.0	38
	Prescription review and reconciliation	Number of blood products dispensed, per 1000 patients discharged	saKPI	4.4	4.3	39
	Prescription review and reconciliation	Number of blood products returned per 1000 patients discharged	saKPI	4.4	4.0	40
	Prescription review and reconciliation	Number of narcotic and psychotropic requests analysed, per 1000 patients discharged	cpKPI	4.3	4.2	41
	Prescription review and reconciliation	Number of narcotic and psychotropic requests dispensed, per 1000 patients discharged	saKPI	4.4	4.0	42
	Outpatient activity	Existence of specific outpatient pharmaceutical consultations (Identify which specialties)	cpKPI	4.8	4.0	43
	Outpatient activity	Number of outpatient pharmaceutical consultations, adjusted by pharmacist FTE	cpKPI	4.5	4.2	44
	Information sharing	Existence of written information regarding prescribed medications at discharge (yes / no)	cpKPI	4.8	4.0	45
	Information sharing	Existence of written information regarding outpatients prescribed medications (yes / no)	cpKPI	4.8	4.0	46
V. Patient safety and quality assurance	The seven rights (patient, medication, dose, route, time, information and documentation)	Existence of inpatient pharmacokinetic monitoring protocols (yes / no)	cpKPI	4.8	4.7	47
	The seven rights (patient, medication, dose, route, time, information and documentation)	Number of inpatient pharmacokinetics monitorizations, adjusted by pharmacist FTE	cpKPI	4.4	4.2	48
	The seven rights (patient, medication, dose, route, time, information and documentation)	Rate of inpatient medicines in clinical pharmacokinetics (which ones)	cpKPI	4.5	4.2	49
	The seven rights (patient, medication, dose, route, time, information and documentation)	Existence of outpatient pharmacokinetic monitoring protocols (yes / no)	cpKPI	4.8	4.7	50
	The seven rights (patient, medication, dose, route, time, information and documentation)	Number of outpatient pharmacokinetics monitorization, adjusted by pharmacist FTE	cpKPI	4.4	4.1	51
	The seven rights (patient, medication, dose, route, time, information and documentation)	Rate of outpatient medicines in clinical pharmacokinetics (which ones)	cpKPI	4.5	4.2	52
	The seven rights (patient, medication, dose, route, time, information and documentation)	Rate of Serum therapeutic concentrations levels in total concentration levels	cpKPI	4.8	4.2	53
	The seven rights (patient, medication, dose, route, time, information and documentation)	Antibiotic use rate (3 classes) in Defined Daily Dose (DDD), per 1000 discharged patients	cpKPI	4.3	4.0	54
	Strategies to identify and reduce errors	Existence of a process that ensures medication batch traceability in outpatient care (yes / no) (which one)	saKPI	4.8	4.5	55
	Strategies to identify and reduce errors	Existence of a process that ensures traceability of all medication batches on inpatient care (yes / no) (which one)	saKPI	4.8	4.5	56
	Strategies to identify and reduce errors	Existence of a process that ensures chemotherapy medication batch traceability (yes / no) (which one)	saKPI	4.8	4.5	57
	Strategies to identify and reduce errors	Existence of a process that ensures blood product medication batch traceability (yes / No) (which one)	saKPI	4.8	4.5	58
	Strategies to identify and reduce errors	Number of internal clinical audits	saKPI	4.7	4.0	59
	Strategies to identify and reduce errors	Number of external clinical audits	saKPI	4.5	4.3	60
	Strategies to identify and reduce errors	Rate of nonconformities in total number of internal audits	cpKPI	4.5	4.0	61
	Strategies to identify and reduce errors	Rate of nonconformities in total number of external audits	cpKPI	4.5	4.0	62
	Monitoring and reporting of adverse events	Number of adverse events reported to National Pharmacovigilance System, per 1000 patients discharged and per number of outpatients followed	cpKPI	5.0	5.0	63
	Monitoring and reporting of adverse events	Rate of patients with medication errors (reported events), per 1000 patients discharged	cpKPI	5.0	4.5	64
	High-risk drug management	Existence of an active pharmacovigilance system (yes / no)	cpKPI	5.0	4.3	65
	High-risk drug management	Number of active pharmacovigilance follow-ups performed	cpKPI	4.6	4.3	66
	High-risk drug management	Number of notifications to the National Pharmacovigilance System that results from active pharmacovigilance, adjusted by pharmacist FTE	cpKPI	5.0	5.0	67
	High-risk drug management	Existence of a list of high-risk medications (yes / no)	saKPI	4.2	4.3	68
	High-risk drug management	Percentage of medications stored according to the LASA (Look-Alike, Sound-Alike) nomenclature	saKPI	5.0	4.0	69
	High-risk drug management	Compliance Index of the last audit according to the LASA (Look-Alike, Sound-Alike) nomenclature	saKPI	4.8	4.0	70
	High-risk drug management	Existence of active medication-related information (yes / no)	cpKPI	4.7	4.5	71
VI. Education and research	Education	Number of undergraduate trainees, adjusted by pharmacy FTE	n.a	4.3	4.0	72
	Education	Number of undergraduate trainees, adjusted by pharmacist FTE	n.a	4.3	4.0	73
	Education	Number of postgraduate trainees, adjusted by pharmacy FTE	n.a	4.3	4.0	74
	Education	Number of postgraduate trainees, adjusted by pharmacist FTE	n.a	4.3	4.0	75
	Continuing education	Time spending on training, adjusted by pharmacy FTE	saKPI	4.2	4.0	76
	Continuing education	Time spending on training, adjusted by pharmacist FTE	saKPI	4.2	4.0	77
	Research and publications	Number of national peer-reviewed publication, adjusted by pharmacist FTE	n.a	4.5	4.0	78
	Research and publications	Number of international peer-reviewed publication, adjusted by pharmacist FTE	n.a	4.7	4.0	79
	Clinical trials participation	Number of clinical trials involving hospital pharmacists	cpKPI	5.0	4.5	80
	Clinical trials participation	Number of clinical trials involving hospital pharmacists, adjusted by pharmacist FTE	cpKPI	5.0	4.5	81
	Clinical trials participation	Number of new clinical trials involving hospital pharmacists, adjusted by pharmacist FTE	cpKPI	4.8	4.5	82
	Clinical trials participation	Existence of a standardized process for implementation and follow-up of clinical trials	saKPI	5.0	4.5	83
	Clinical trials participation	Number of patients in clinical trials in which the pharmacist is involved, adjusted by pharmacist FTE	cpKPI	4.8	4.3	84
	Clinical trials participation	Experimental medication dispensing error rate	cpKPI	5.0	4.0	85

*EAHP* European association of hospital pharmacists, *saKPI* support activity key performance indicator, *cpKPI* clinical pharmacy key performance indicator, *FTE* full time equivalent, *n.a.* not applicable, Relevance and Measurability scores ranged from 1–5 points

## Discussion

Although being the first study to define a Portuguese set of saKPIs/cpKPIs to assess hospital pharmacy performance and quality based on EAHP Statement, other countries have deployed similar studies, as previously mentioned.

Though some authors argue that setting benchmarks by accreditation bodies or certifications by international organizations is the first step of healthcare quality cycle [49], only one study from Brazil referred to the importance of measuring the existence of updated written operational procedures for all clinical pharmacy activities [34].

About the KPIs included in the second statement, the ‘*Drugs stock turnover*’ comprises a commonly used criterion for assessing the efficiency of pharmacies’ purchasing and supply chain [26]. Given the importance of this metric, three indicators were defined in the Magarinos-Torres et al*.* (2007) study to assess the stock turnover, namely the number of medication units lost, the value spent in lost medication, and the existence of updated reports on medication availability [34].

Our expert panel included the highest number of KPIs in the “clinical pharmacy services” statement, which is aligned with the indicators referred across the literature. The KPI ‘*Existence of medication reconciliations up to 72 h after admission*’ is one of the most frequently mentioned in the literature [4, 5, 17, 19, 26, 45]. Although the time interval defined for reconciliation varies between 24 to 72 h, there is a consensus across the literature that it is highly relevant and measurable, not only by identifying the existence or non-existence of these reconciliations, but also by the importance of quantifying the proportion of patients who received formal documented medication reconciliation at discharge [4, 26].

Aligned with several studies [4, 19, 26, 45], KPIs related to patients’ education and information sharing are highly recommended. In the Lloyd et al*.* (2017) study, the expert panel argues that patients have to receive written/verbal counselling before discharge, and that they should also receive a document with an accurate medication list detailing any therapy changes [26]. In two other studies, both panel groups defined a specific KPI to measure the proportion of patients who have face-to-face discussions about their medication before discharge [4, 45].

Concerning the c*linical pharmacy services*, the development of outpatient pharmaceutical consultations was considered an important area of clinical intervention, aiming for medication reconciliation, drug interactions management, adverse reactions detection, patient education among others [50–52]. Therefore, outpatient pharmacy and consultation has become an important part of pharmacists’ tasks [52, 53].

As for the statement *patient safety*, although the number of adverse events reported by staff and/or by patients is one of the KPIs most frequently referred to in the literature, this indicator can assume different definitions. For example, in a study from Brazil, this KPI is mentioned as ‘*Number of problems that occurred related to medications*’ and ‘*Number of problems related with medications identified and notified*’ [34]; in a study from Belgium, it’s referred to as ‘*Number of interventions accepted and activities performed to prevent, detect, assess, manage, report, and/or document adverse drug reactions/Number of patients with a pharmaceutical record*’ [19]. Given that the Portuguese National Pharmacovigilance System requires pharmacists to report adverse events, the expert panel included a specific KPI to quantify the ratio between reported adverse events and number of discharges/ outpatients followed.

Finally, all panel members agreed that pharmacist’s continuous education was one of the key elements to ensure the quality of care and should therefore be assessed. Similarly, an Australian study concluded that continuous training is part of the learning process of clinical pharmacists in 239 public hospital pharmacy services [7]. Thus, it is not surprising that the cpKPI ‘*Time spent on training*’ was considered relevant by our expert panel, as well as by several studies [26, 34, 38]. According to this last study, participation in continuing long-term higher education usually includes areas such as expertise in ward pharmacy, medication reviews, or accreditation for comprehensive medication reviews [38].

The key outcome of this study was the definition of the first list of national KPIs to assess hospital’s pharmacists’ performance. Additionally, the study design enabled an important advantage, given the characteristics of the expert panel, which included individuals who feel more comfortable participating in face-to-face meetings rather than in multi-round surveys. The use of a combined nominal group/focus group technique ensured equal participation amongst panellists and gave them the opportunity to explore diverging opinions throughout the group discussions. Finally, when implemented at the national level, we expect that these KPIs will improve transparency and accountability among hospital pharmacies and heighten the quality of care.

As for potential weaknesses, the exclusion of relevant KPIs by the expert panel based on the low measurability may limit the scope of assessment. However, since the indicators included in the final list reflect the dichotomy between high relevance and measurability, it is expected that its adoption by hospital pharmacies and by the Ministry of Health may become a reality before long.

## Conclusion

Defining these 85 *cpKPIs/saKPI* is a first step towards assessing Hospital Pharmacy performance and quality. Major challenges are expected to arise during the implementation of these KPIs at a national level. Some of which include: defying the *status quo*, increasing workload in data collection, ensuring data quality and, most importantly, communicating across all players that KPI measurement to monitor performance in hospital pharmacists’ clinical and support activities will allow better activity assessment, leading to an improvement in inpatient and outpatient quality of care, enabling continuous future development and planning with greater certainty.

Despite this study’s major contribution to hospital pharmacists’ clinical and support activities, future research should focus on gathering external stakeholders’ feedback on relevant KPIs, developing consensus indicators for outpatient care or for subspecialty areas, which require different and/or supplemental metrics to help improve quality of patient care and further develop clinical pharmacy practice. Thus, future research ought to contribute to a more complete understanding of KPIs role in this field.
